# Phylogenetic signal in floral temperature patterns

**DOI:** 10.1186/s13104-021-05455-5

**Published:** 2021-01-28

**Authors:** Sean A. Rands, Michael J. M. Harrap

**Affiliations:** School of Biological Sciences, University of Bristol, Life Sciences Building, Tyndall Avenue, Bristol, BS8 1TQ UK

**Keywords:** Thermography, Flowering plants, Temperature patterns, Pollinator-flower interactions, Phylogenetic signal

## Abstract

**Objectives:**

Floral structures may be warmer than their environment, and can show thermal patterning, where individual floral structures show different temperatures across their surface. Pollinators can differentiate between artificial flowers that mimic both naturally warmed and thermally patterned ones, but it has yet to be demonstrated that these patterns are biologically meaningful. To explore the relationship between pollinators and temperature patterning, we need to know whether there is diversity in patterning, and that these patterns are not simply a by-product of floral architecture constrained by ancestry. We analysed a dataset of 97 species to explore whether intrafloral temperature differences were correlated within clades (phylogenetic signal), or whether the variation seen was diverse enough to suggest that floral temperature patterns are influenced by the abiotic or pollinator-related niches to which plant species are adapted.

**Results:**

Some phylogenetic signal was observed, with both the Asteraceae and species of *Pelargonium* being more similar than expected by chance, but with other species surveyed not showing signal. The Asteraceae tend to have large temperature differences across the floral surface, which may be due to floral architecture constraints within the family. Other families show no correlation, suggesting that patterning is influenced by pollinators and the environment.

## Introduction

Many plant species rely on animal pollinators to transfer pollen between individuals to ensure successful reproduction [1–3]. To attract pollinators, plants produce floral displays that may be detectable by the pollinator across many sensory modalities, including colouration [4–9], scent [10, 11], tactile structure [12, 13], electrostatic charge [14, 15], orientation [16, 17], polarisation of light [18], and humidity [19, 20]. Floral temperature may be another signalling mode. Flowers are frequently warmer than their surrounding environment through the absorption and retention of solar radiation [21–25], and many floral features exist that aid in maintaining a thermal microenvironment within the flower [26]. The temperature differences seen are potentially biologically meaningful, as similar differences in the temperature of artificial flowers have been demonstrated to be detectable by bumblebees [27, 28], honeybees [29] and stingless bees [30]. Given that pollinators can detect the temperature differences produced, understanding the role of floral temperature as a potential cue to pollinators is important if we are to understand whether and why flowers produce such complicated multimodal displays [31–33].

Patterning of a display can further enhance the pollinator response, both by increasing detection and learning speed [7, 34–36], and by increasing the speed of performance once the pollinator is on the flower, through the use of nectar guides [37–42]. Patterning is not limited to visual stimuli, and different regions of a flower may show different texture [12], scent [43–47], or temperature [23–25, 48, 49]. Harrap et al. [48] demonstrated that these intrafloral differences in temperature are regularly patterned in many plant species (55% of 118 surveyed), where the different regions of an individual flower could differ in temperature sufficiently for a visiting pollinator to be able to detect this difference (where the mean temperature difference within a flower was 4.9 °C). This study also demonstrated that the form of temperature patterning can differ between species, and that temperature patterns can occur across flowers showing radial and bilateral symmetry, often leading to similarly symmetrical temperature patterns (Additional file 1: of [48]). Experimental tests [48] demonstrated that bumblebees can learn to differentiate between artificial flowers with differing temperature patterns, and this learning can be enhanced when a colour cue is added as a multimodal co-signal [50].

Given both that floral temperature patterns exist and differ between plant species, and that pollinators can learn and respond to artificially-produced patterns presented at a biologically-meaningful thermal scale, we now need to explore how these patterns influence pollinators in the wild, and understand whether interspecific patterning differences affect signal efficacy or the response of the pollinator. To do this, we first need to identify whether there is interspecific variation in the patterns produced. Any variation identified could suggest that the patterns produced are not merely a by-product of floral architecture, but are either a result of interactions with the environment or an adaptation for pollinator attraction.

Here, we test whether one aspect of temperature patterns is constrained by the evolutionary history of a plant, by considering the magnitude of the intrafloral temperature differences across a taxonomically diverse sample of species. By looking for phylogenetic signal (in the sense of [51]), we can identify whether the measurements made on any lineage are more correlated than we would expect by chance, which would demonstrate evolutionary constraint. Temperature differences may also be constrained by the shape of the flower and pattern, and we test this by examining whether a crude measure of shape, based on floral symmetry, can be used to predict mean temperature difference.

## Main text

### Methods

We used data previously reported in Additional file 1: of [48], which collected thermal images of the floral displays (flowers or where more appropriate inflorescences) of 118 species on sunny days in sunlight, following accepted thermography protocols [52], and intrafloral temperature differences were measured from these images. Species were recorded from three UK (temperate) botanic collections and were selected with the aim of sampling flowers visited by a wide range of floral visitor groups and as broad range of floral shapes, colours and phylogeny as possible, rather than focussing on species where low environmental temperatures and floral ecology may influence floral temperature (*e.g. * [21, 53, 54]*.*). The dataset was reduced by removing species represented by cultivars or multiple colour morphs, and ambiguous unidentified species where only the genus name was given. This left 97 species from 39 families. Each species had a ∆temp value associated with it, representing the temperature difference between the warmest and coldest part of a single individual flower (or inflorescence where more appropriate), with ∆temp ranging from 0.0 to 11.8 °C. Full details of how ∆temp was surveyed and recorded, as well as details on the environmental conditions during sampling, are given in [48] (plus see the Limitations below).

All analyses were conducted using *R* 3.6.2 [55] (code and data are presented in Additional files 2 and 3). A dichotomised phylogeny was constructed using scenario 3 of *V.Phylomaker* [56], and converted to phylo4d format using *phylobase* 0.8.10 [57]. Phylogenetic signal was measured using *phylosignal* 1.3 [58]. Because we were not making assumptions about trait evolution, we used Moran’s *I* to measure phylogenetic autocorrelation [59–61]. Because autocorrelation was detected in the dataset, we then explored the dataset using a *Local Indicator of Phylogenetic Association* (*LIPA*) analysis to identify where species were autocorrelated with neighbouring species, by calculating local Moran’s *I* (*I*_*i*_) [62], using the *LIPA* technique outlined by [58].

The data were classified in Additional file 1: of [48] as showing either radial or bilateral symmetry. This classification was based on the symmetry shown by the flower: ‘Radial’ flowers showing symmetry about a central point; ‘Bilateral’ flowers showing symmetry about a single plane. Whether ∆temp was related to floral symmetry was tested using a phylogenetic ANOVA [63], using the *phylANOVA* function within *phytools* 0.7–20 [64].

## Results

**Fig. 1:**
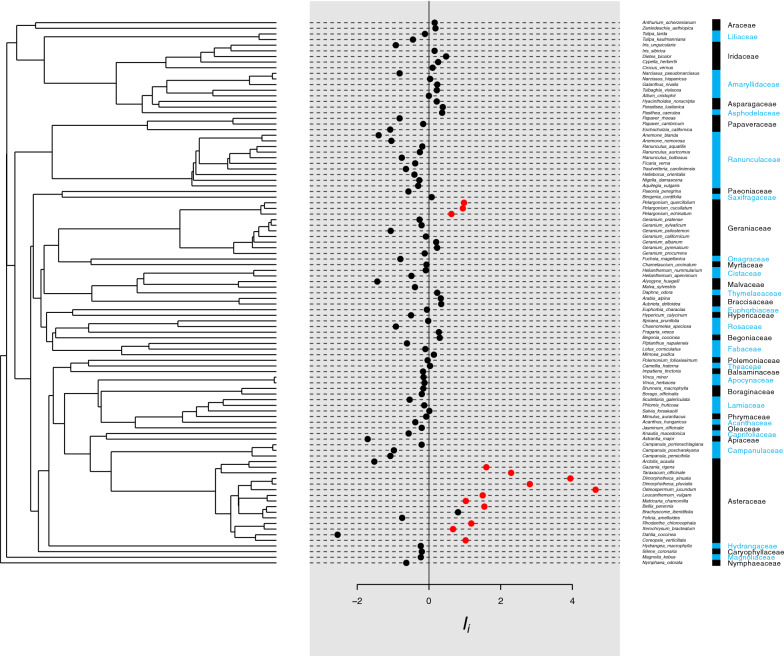
Local Moran’s index (*I*_*i*_) values for each species’ measured temperature difference, together with the phylogeny used. The value of *I*_*i*_ denotes the degree of autocorrelation between closely-related species, and each value calculated has a corresponding significance associated with it: red dots denote significant *I*_*i*_ values, where *p* < 0.05. The figure demonstrates that significant local autocorrelation is concentrated within *Pelargonium* and the Asteraceae, and these significant values of *I*_*i*_ are positive, meaning that these species are positively autocorrelated (*e.g.* more similar to each other than would be expected due to random processes)

A phylogenetic signal was detected (*I* = 0.056, *p* = 0.005). The *LIPA* analysis showed that there was significant local positive autocorrelation in two clades: the *Pelargonium* genus within the Geraniaceae, and within the Asteraceae (the clump of largely positive *I*_*i*_ values towards the bottom of Fig. 1).

Since there was some phylogenetic signal within the data, it was appropriate to conduct a phylogenetic ANOVA. This showed that maximum temperature difference was not related to the symmetry of the flower and floral heating (*F*_1,95_ = 3.64, *p* = 0.188).

## Discussion

The data demonstrate that there is relatively little phylogenetic signal in the temperature differences within floral temperature patterns in the 97 species surveyed, suggesting that this trait is unlikely to be constrained by evolutionary history [51, 58]. Since the species were sampled under similar environmental conditions (taken from three managed garden collections within 130 km of each other), this suggests that the temperature differences within the patterns are determined by either the life history and selection, environment of the plant, or by the interaction with pollinators, or else the difference within the patterning is random and not subject to selection. Disentangling the first two functional explanations from the final null reason requires further study: an exploration of the relationship of patterning with the environment would confirm the first of these reasons, whilst further experimentation to confirm that temperature patterns in real plants can influence pollinator behaviour would add support to the second reason. Floral thermal biology is influenced by a wide range of morphological, physiological and environmental factors [24, 26, 48, 54, 65], and so we recommend that attention is paid to taxonomically distant sympatric plant species which share one or a few important generalist pollinator species.

Although most species surveyed showed no phylogenetic signal, we observed that temperature differences in the Asteraceae and the three *Pelargonium* species that we examined were more similar than would have been expected by chance, suggesting that this difference was at least partially constrained by shared recent history. Although the survey contained a large number of Asteraceae, we did not see a similar signal in other clades with large samples such as the Ranunculaceae. Similarly, although the three *Pelargonium* sampled were closely related within the same genus, we did not observe any signal in the larger sample of *Geranium* species, which are within the same family (Geraniaceae) as *Pelargonium*.

The data presented in [48] show that large temperature differences are seen within the floral displays of the Asteraceae, while the *Pelargonium* species tended to show very little temperature difference across their flowers. Floral architecture may explain these correlations in intrafloral temperature differences, at least in the Asteraceae. Compound inflorescence (capitulum) architecture is maintained across the Asteraceae [66], and this shared architecture may lead to a common warming response to the environment. The tightly-packed florets at the centre of compound inflorescences lead to a high surface area relative to the petals of ray florets that make up the floral display’s periphery. The disc florets of the Asteraceae are also often a darker colour than the periphery of the ray petals. This means disc florets heat up faster in sunlight than ray petals, and dense packing of disc florets may aid heat retention. The influence of floral architecture in floral warming of the Asteraceae is demonstrated by the common shape of temperature patterns that align with inflorescence structure in the Asteraceae [24, 25, 48, 54], with disc florets being warmer, and ray petals cooler. While other clades have a conserved floral structure in common across species, it is possible that these do not predispose flowers to warm consistently in certain way or to a similar degree. It is unclear if floral structure explains the correlation in *Pelargonium*. However, flower orientation can influence a flower’s ability to capture sunlight for warming [22, 25], and it is possible that the fixed horizontal floral orientation shared by these *Pelargonium* species limits their ability to warm.

Our results also show that floral symmetry, which was assumed to relate to temperature pattern shape, does not correlate with the size of the temperature difference shown within a pattern, which again suggests that the environment and pollinators that a plant is adapted for may be important. The dichotomous classification of temperature patterns into bilateral or radial presented in [48] is admittedly a crude measure based on an easily available selection of species with larger flowers that are accessible to thermography, and we would suggest that a more detailed survey is conducted in order to identify the range of patterns that exist and whether it is possible to classify them.

Heat may only be one of the many cues and signals that multimodal flowers can present to their pollinators [1, 31, 32, 50, 67], but understanding how a possible heat cue can influence pollinator behaviour is important given current concerns about climate change. Small environmental temperature changes could alter the floral thermal microhabitat sufficiently to influence the behaviour of visiting pollinators [65], which could have subsequent effects on plant fitness. As well as being a cue, floral temperature could also act as an energetic reward for a pollinator by allowing them to passively elevate their body temperature [21, 68], and both changes in the temperature of the local environment and changes to rainfall due to climate change [69] could have similar impacts on both plant and pollinator fitness. If small environmental temperature changes can affect how effective a warm flower is at influencing pollinator behaviour, it is important that we understand whether intricate patterns of intrafloral temperature difference are a vital component of pollinator attraction before climate change renders them irrelevant to their target pollinators.

## Limitations

The intrafloral temperature differences used are based on single observations of each species on sunny days while in direct sunlight. These observations do not consider the potential variation in temperature patterns that may exist within flower species. Environmental conditions during thermal imaging were not rigorously monitored. It is possible intrafloral temperature differences are affected by differences in environmental conditions potentially obscuring correlations between species.

It is uncertain that all the species measured would have given temperature differences within their natural range, as their flowers might heat up differently in native conditions compared to botanical collections used here. In managed gardens, related species are often grown together, so correlation may result from species being imaged concurrently in similar environmental conditions. Here, *Pelargonium cucullatum* and *P. echinatum* were imaged immediately after each other, but *P. quercifolium* was imaged a year prior and still showed a correlating low ∆temp. Similarly, fifteen Asteraceae species were sampled on seven separate days. Thus, while timing effects may explain correlation in the smaller *Pelargonium* group it is unlikely to contribute to correlation in Asteraceae.

## Supplementary Information

**Additional file 1.** Comma delimited data file, reformatting the used subset of the data originally presented in [48].

**Additional file 2.** R code file describing analysis (opens as a text file).

**Additional file 3.** Text file presenting temperature data reordered for use with *phylo4d* data structures.

## Data Availability

The dataset processed for this article is freely available as described by [48] at https://doi.org/10.7554/eLife.31262.009. The *R* Code and processed datasets are available as additional material to this manuscript.
